# Visage sacro-iliaque percutané pour fracture instable du bassin de type C 1-2: à propos d'un cas au CHU de Yaoundé

**DOI:** 10.11604/pamj.2014.19.292.5507

**Published:** 2014-11-17

**Authors:** Marc Leroy Guifo, Ibrahima Farikou, Christopher Tagnyin Pisoh, Aurélien Ndoumbé, Serge Blaise Emaleu, Samuel Takongmo

**Affiliations:** 1 Chirurgie Générale et Orthopédique CHU de Yaoundé, Faculté de Médecine et Sciences Biomédicales, Université de Yaoundé 1, Cameroun; 2 Chirurgie orthopédique Centre National de Réhabilitation des Handicapés (CNRH), Yaoundé; 3 Neurochirurgie, CHU de Yaoundé, Faculté de Médecine et Sciences pharmaceutiques de Douala, Cameroun; 4 Senior Clinical research scientist herzenberg laboratory, Beckman Center, Department of Genetic/Immunology, Standford University School of Medicine, California

**Keywords:** fracture pelvienne instable, ostéosynthèse percutanée, fluoroscopie, Unstable pelvic fractures, percutaneous osteosynthesis, fluoroscopy

## Abstract

Les ruptures instables de l'anneau pelvien nécessitent une prise en charge chirurgicale. Elles ont traditionnellement été traitées par des ostéosynthèses à foyer ouvert ou par une prise en charge non opératoire lorsque les compétences n’étaient pas disponibles. Il en découlait des séquelles douloureuses et gênantes pour les patients. En 1993 Routt et coll ont rapporté la technique du vissage sacro-iliaque percutané basée sur l'utilisation de la fluoroscopie. Cette technique a été adoptée dans les pays avancés, mais aucune publication ou utilisation n'a été faite à notre connaissance dans notre milieu. Nous rapportons ici un cas réalisé au CHU de Yaoundé et discutons des considérations médicales et techniques qui en découlent.

## Introduction

Le traitement chirurgical des fractures instables du bassin présente des difficultés de réduction et de contention [1]. Il est souvent à la charge de quelques praticiens dans les centres spécialisés qui ont une expertise significative pour être entretenue et transmise par l'enseignement. Cette nécessité s'est imposée de par la rareté des indications opératoires, l'exigence d'une bonne connaissance de l'anatomie nécessaire aux voies d'abords. L'assistance d'un chirurgien généraliste est souvent recommandée [2]. Dans les centres moins équipés, ces traitements peuvent à priori apparaître inaccessibles du fait de l'absence d'expertise confirmée. Les traitements orthopédiques non opératoires sont entrepris par défaut car souvent suivis de séquelles fonctionnelles à type de douleurs et de boiteries. Dans la série de référence de Holdsworth, 15 patients sur 27 n'ont jamais pu reprendre une activité professionnelle [1, 3]. L'introduction des techniques percutanées, le traitement informatique des images notamment avec reconstruction au scanner, et le perfectionnement de l'instrumentation orthopédique avec les vis cannelées, ont paradoxalement rendu le traitement plus aisé moyennant l'investissement raisonnable qu'est l'amplificateur de brillance. Nous rapportons ici un cas au CHU de Yaoundé.

## Patient et observation

**Figure 1:**
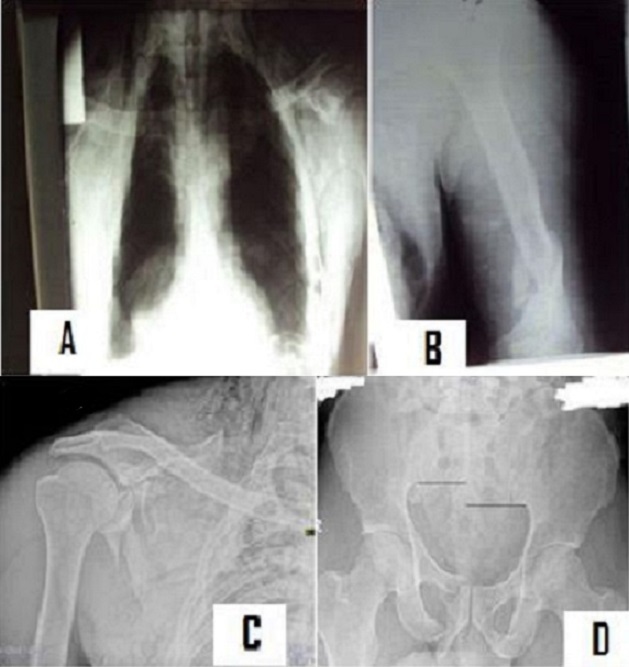
Bilan lésionnel: (A) contusion pulmonaire et hémothorax droit; (B) fracture tiers inférieur de l'humérus gauche; (C) fracture de l'omoplate droit; (D) rupture antérieure et postérieure de l'anneau pelvien Tile C1-2

**Figure 2:**
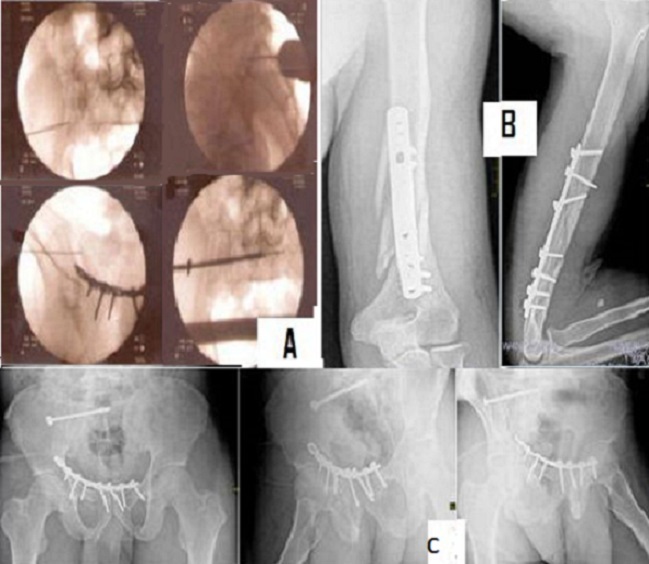
Images per et post opératoires: (A) insertion per opératoire d'une broche guide avec contrôle scopique en vue de face et latérale et mise place de la vis cannelée sur la broche; (B) contrôle post opératoire fracture de l'humérus; (C) contrôle post opératoire de la fracture du bassin

Mr N.O âgé de 33 ans est référé d'un hôpital de la ville de Yaoundé suite à un accident de la voie publique survenu quelques heures auparavant. A l'examen primaire on notait une matité de l'hémothorax droit avec abolition des murmures vésiculaires à la base droite. Il existait une déformation du bras gauche avec une impotence fonctionnelle. Le membre inférieur droit était douloureux à la mobilisation avec des man'uvres de Larrey et Verneuil positives. Le reste de l'examen était sans particularité notamment une absence d'hématurie ou de saignement urétral. Les radiographies initiales ont montrées une fracture comminutive de la diaphyse humérale gauche, une fracture articulaire de l'omoplate droite, une fracture du bassin avec ascension de l'hémi bassin droit classé C1-2, et un hémothorax droit (Figure 1). Sa prise en charge a consisté un drainage thoracique droit, une attelle du bras gauche et une traction collée du membre inférieur droit. La fracture de l'omoplate droite a été traitée par une immobilisation coude au corps dans une écharpe appropriée. La fracture de l'humérus a été traitée par ostéosynthèse par plaque vissée. L'ostéosynthèse des fractures du bassin a été faite sous anesthésie générale, Le patient était installé en position de décubitus dorsal, un billot médian permettant un accès suffisant à la fesse droite jusqu'aux épines iliaques. Le thorax était stabilisé sur la table par des supports latéraux. Cette installation était faite par juxtaposition de table opératoire permettant de situer le bassin du patient sur une zone radio transparente (Figure 2). Pour l'ostéosynthèse du bassin, nous avons eu recourt à un ancillaire spécifique comprenant des vis cannelées, un tournevis cannelé, une broche de 2 mm pour la recherche du trajet, une mèche cannelée, une mèche flexible et un tournevis poly axial permettant l'insertion des vis dans la cavité profonde. Les suites opératoires immédiates ont été simples.

## Discussion

Les fractures du bassin sont des urgences vitales lorsqu'elles entraînent une instabilité de l'anneau pelvien [4, 5]. Elles sont inhérentes à la mobilité des individus imposée par l'activité socioprofessionnelle. Les fractures instables posent des problèmes hémodynamiques lorsqu'elles sont accompagnées de lésions vasculaires et viscérales. Le trajet pelvien des artères et veines iliaques ainsi que leurs nombreuses collatérales donnent une prédisposition anatomique à ces lésions [6]. Ces fractures représentent 5% des fractures et surviennent dans le cadre des polytraumatismes dans 20% [6]. Elles imposent parfois des mesures de stabilisation à la phase primaire comportant un remplissage hémodynamique et une immobilisation du bassin [4]. La fixation externe en urgence par le fixateur de Hoffman, la pose d'un pantalon antichoc ou un clamp pelvien et plus simplement une traction trans-osseuse permettent dans ces cas de comprimer le bassin et ou de stabiliser les caillots permettant ainsi de franchir le cap de l'urgence initiale, puis de procéder aux explorations nécessaires. Dans les suites de cette prise en charge primaire, il est nécessaire de déterminer quels sont les patients qui nécessitent des actes complémentaires d'ostéosynthèse de l'anneau pelvien. La plupart des auteurs opposent les fractures stables aux fractures instables. Un bassin est considéré stable s'il peut subir des contraintes physiologiques sans déformation anormale. L'examen physique notamment les man'uvres de Larrey et Verneuil permettent de dépister une instabilité potentielle. Ces man'uvres sont indispensables lorsque les radiographies initiales comportant les incidences de face et inlet/outlet, n'ont pas décelé des déplacements révélant une instabilité. Il est admis qu'au-delà de 2.5 cm d'ouverture de la symphyse pubienne, il existe une béance de l'articulation sacro-iliaque et une instabilité rotatoire. Plus souvent ce sont les branches ilio et ischio-pubiennes qui auront été fracturées. Le scanner permet mieux que la radiographie standard de faire le bilan lésionnel précis. De nombreuses classifications notamment de Letournel, de Young et Burgess ou de Tile, permettent de classer les fractures ou ruptures de l'anneau pelvien. La classification de Letournel donne une localisation anatomique mais aucun renseignement sur la stabilité. La classification de Young et Burgess s'appui sur le mécanisme lésionnel et distingue les compressions antéro-postérieures, les compressions latérales et les cisaillements. Elle reconnait des lésions associées différentes statistiquement dans les divers groupes, ainsi, les compressions antéro-postérieures sont plus associées à des lésions vasculaires que les compressions latérales qui s'accompagnent volontiers de trauma crânien et lésions viscérales intra-abdominales [3]. La classification de Tile prenant en compte la stabilité des lésions donne un instrument permettant de poser les indications thérapeutiques et d’évaluer les résultats obtenus. Nous avions donc une facture classé C1-2 selon la classification de Tile car il existait sur le cliché initial une ascension de hémi bassin droit (Figure 1). La prise en charge de ces fractures a traditionnellement été conservatrice en raison de difficultés anticipées d'une chirurgie invasive et d'une focalisation sur la survie initiale des patients [5].

L'histoire naturelle de cette prise en charge a été mieux connue avec les travaux des auteurs comme Tile sur des séries importantes de patients [1]. La persistance des douleurs lors de la déambulation, la boiterie, les pseudarthroses constituent les complications les plus rencontrées et sont majorés par le traitement non opératoire [5]. Ces complications sont d'autant plus fréquentes que la réduction a été insuffisante. Pour Letournel, la réduction parfaite des fractures du bassin est indispensable mais n'est pas toujours réalisable en raison de la gravité des lésions et du délai parfois nécessaire pour la stabilisation du polytraumatisé. Il n'est pas prouvé par ailleurs qu'un bassin consolidé avec un déplacement mineur (inférieur à 2 cm) soit moins confortable qu'un bassin réduit anatomiquement. Les seuils exigibles restent ainsi à définir. La fixation définitive de l'anneau vise la restitution de la solidité de la charnière sacro-iliaque et utilise des moyens divers tels que la fixation externe, la plaque postérieure ou la fixation endo-pelvienne par des plaques vissées après un abord ilio-inguinal selon Judet Letournel. Cette fixation postérieure peut être associée à une fixation de l'arc antérieur pour une restitution de l'anatomie et pour contribuer la stabilité de l'ensemble de l'anneau. La fixation du bassin après l'abord de judet Letournel est difficile de par la profondeur, la complexité de l'anatomie, le voisinage des vaisseaux iliaques ainsi que des racines nerveuses L5 et S1. Ces difficultés ont rendu toute initiative d'intervention périlleuse à priori d'autant que les complications de ces abords sont réputés plus importantes que celle du traumatisme avec un taux de surinfection pouvant atteindre 27% [7]. Le vissage sacro-iliaque est une technique qui a été rapportée depuis 1993 par Routt et collaborateurs. Cette technique a été améliorée par l'utilisation des vis cannelées insérées avec une instrumentation appropriée sur une broche-repère préalablement mise sous contrôle scopique, sur un trajet ilio-sacré passant dans le pédicule de S1 ou S2. Cette technique apparait sécurisée par la marge offerte sur le diamètre du pédicule de S1 de 28 mm, comparée au diamètre de la broche guide de 2 mm. L'utilisation de la scopie en per-opératoire permet le contrôle du trajet bien avant le franchissement de l'articulation sacro-iliaque, et la possibilité de faire plusieurs tentative dès lors [3]. Nous avons trouvé l'utilisation de l'incidence de profil appropriée pour repérer le pédicule S1 avant le contrôle par les autres incidences inlet et outlet, minimisant les risques pour l'artère fessière et le nerf fessier [8, 9]. Cette technique impose une réduction anatomique parfaite seule garante des repères et d'une bonne analyse des images de fluoroscopie. Le taux rapporté de lésions vasculo-nerveuses est de 18% selon les études disponibles, ce qui semble élevé par rapport aux risques estimés et incite à plus d'attention [7]. Il est recommandé de procéder à une réduction ouverte en cas de réduction insuffisante et de fixer au choix par la même technique ou des plaques vissées. Cette exigence pour Rommens devrait inciter à ce que seuls les chirurgiens capables de faire une ostéosynthèse à foyer ouvert entreprennent l'opération percutané [10]. Notre patient avait été mis en traction initialement, ce qui nous semble avoir contribué à la réduction de la fracture (Figure 2). La fixation percutané par vis cannelé est également pratiquée pour les fractures de branches ilio-pubiennes [10]. Elle pourrait ainsi augmenter l'intérêt de la fixation chirurgicale pour les patients nécessitant une mobilisation rapide comme les athlètes, et pour minimiser les risques de dystocies en obstétrique.

## Conclusion

En conclusion le vissage percutané des fractures sacro-iliaques nous semble une technique peu invasive, praticable chaque fois que les circonstances le permettent, d'autant plus que ces fractures surviennent dans un contexte de polytraumatisme parfois avec d'autres lésions pouvant mettre en jeu le pronostic vital.
